# Attitudes Towards Non-directiveness Among Medical Geneticists in Germany and Switzerland

**DOI:** 10.1007/s11673-024-10355-x

**Published:** 2024-07-22

**Authors:** J. Eichinger, B. S. Elger, S. McLennan, I. Filges, I. Koné

**Affiliations:** 1 Institute for Biomedical Ethics, University of Basel, Bernoullistrasse 28, 4056 Basel, Switzerland; 2 Center for legal medicine (CURML), University of Geneva, Rue Michel-Servet 1, 1211, 4 Geneva, Switzerland; 3 Institute of History and Ethics in Medicine, TUM School of Medicine, Technical University of Munich, Ismaninger Straße 22, 81675 Munich, Germany; 4 Medical Genetics, Institute of Medical Genetics and Pathology, University Hospital Basel and University of Basel, Schönbeinstrasse 40, 4056 Basel, Switzerland; 5 Department of Clinical Research, University Hospital Basel and University of Basel, c/o Universitätsspital Basel, Spitalstrasse 8/12, 4031 Basel, Switzerland

**Keywords:** Medical ethics, Clinical decision-making, Genetic counseling, Personal autonomy, Directive counseling, Decision making, Shared, Physician–patient relations, Patient-centered care

## Abstract

The principle of non-directiveness remains an important tenet in genetics. However, the concept has encountered growing criticism over the last two decades. There is an ongoing discussion about its appropriateness for specific situations in genetics, especially in light of recent significant advancements in genetic medicine. Despite the debate surrounding non-directiveness, there is a notable lack of up-to-date international research empirically investigating the issue from the perspective of those who actually do genetic counselling. Addressing this gap, our article delves into the viewpoints and experiences of medical geneticists in Germany and Switzerland. Twenty qualitative interviews were analysed employing reflexive thematic analysis. Participants’ responses revealed substantial uncertainties and divergences in their understanding and application of the concept. It seems to cause distress since many geneticists stated that the principle was difficult to put into clinical practice and was no longer ethically justified given the increasing likelihood of therapeutic implications resulting from genomic testing outcomes. The insights provided by our qualitative empirical study accord with the ongoing theoretical debate regarding the definition, legitimacy, and feasibility of the principle. An adequately nuanced understanding and application of non-directiveness seems crucial to circumvent the risks inherent in the principle, while promoting patient autonomy and beneficence.

**Supplementary Information:**

The online version contains supplementary material available at 10.1007/s11673-024-10355-x.

## Introduction

The principle of non-directiveness remains an important tenet in genetics, in guidelines of various professional societies (Manickam et al. 2021; S2k-Leitlinie 2019) and, for example, in Switzerland even in the law (GUMG 2022). Although there is no consensus on a definition of the principle, non-directiveness typically refers to “an approach to genetic counselling that aims not to guide the patient^1^ (or client) to an outcome predetermined by the counsellor or the genetics service but instead to support the patient in reaching their own decisions” (Clarke 2017, 543). Historically, non-directiveness was originally established in the United States at the end of the 1960s and internationally in the 1980s, with the aim of promoting patients’ autonomous decision-making—by then primarily understood as non-interference (Kessler 1992; Jamal, Schupman, and Berkman 2020). Another significant objective was to distance the field from paternalistic medicine in general. Additionally, the concept of non-directiveness emerged as a response to disassociate from eugenic practices and ideologies prevalent in Europe and the United States during the first half of the twentieth century (Clarke 2017). Other contributing factors were the growing recognition of women’s rights, including in the field of reproduction (Chańska 2022).

However, the principle of non-directiveness in genetic counselling has faced increasing criticism over the last two decades (Weil et al. 2006). There is an ongoing discussion about its appropriateness in genetics and whether it might be more appropriate or less suitable in certain cases. It is argued, for example, that the underlying notion of autonomy is overly narrow, risking a conflation of non-directiveness with neutrality (Clarke 2017). Critics suggest that non-directiveness may hinder the establishment of a therapeutic alliance (Biesecker, Peters, and Resta 2019). Furthermore, it is suggested that autonomy should rather be understood relationally and that autonomy should be balanced more appropriately against other vital ethical principles such as beneficence or non-maleficence (Jamal, Schupman, and Berkman 2020). Another reason mentioned is that the conceptual and also practical landscape around genetic counselling and clinical genetics has evolved considerably since the uptake of the non-directiveness, with genetic testing now playing an increasing role for treatment decisions and prevention (Elwyn, Gray, and Clarke 2000; Gyngell et al. 2019; Schupman, Jamal, and Berkman 2020; Clarke 2023). Critics also contend that non-directiveness is unattainable in practice because it is impossible to impede all unintentional or non-verbal expressions of the counsellor’s opinion and because shaping factual information, especially constructing risk statements, always conveys values (Bosk 1993; LeRoy 1993; Caplan 1993; Clarke 2019; Chańska 2022). These concerns have led to some newer guidelines omitting the use of term non-directiveness (Vears et al. 2021; Chańska 2022).

Despite the debate surrounding the concept of non-directiveness, apart from one study published in 2022 with Polish medical geneticists (Chańska and Grunt-Mejer 2022), we are not aware of any up to date international research that empirically examines the issue from the perspective of those who actually do the genetic counselling; since studies that were published in 1997 (Bartels et al. 1997; van Zuuren et al. 1997) now have limited value due to the significant developments that the field of genetics has undergone in recent years. Furthermore, the majority of the existing literature on non-directiveness originates from major English-speaking countries, while cultural interpretations of autonomy—which are inextricably linked to the concept of non-directiveness—vary significantly. This article, therefore, aims to explore the views and experiences of medical geneticists in Germany and Switzerland regarding the principle of non-directiveness, which were brought up in qualitative interviews exploring ethical considerations in paediatric genome-wide sequencing more generally as part of a larger study. Although the interviews actually took place in the context of diagnostic paediatric genome-wide sequencing, many geneticists consistently brought up other genetic counselling situations on their own initiative, such as those in the prenatal context.

## Materials and Methods

The study methods adhere to the Consolidated Criteria for Reporting Qualitative Research (COREQ) reporting guideline (Tong, Sainsbury, and Craig 2007). See supplement 1 for a detailed description of the methodology.

### Research Team and Reflexivity

Interviews were conducted by the PhD student in biomedical ethics J.E. Most of the researchers involved have long-established experience with qualitative research. They have backgrounds in Bioethics (J.E., B.E., S.ML., I.K.), Medicine/Medical Genetics (B.E., I.F., I.K.), Political Science (J.E.), and Theology (B.E.). One of the interview partners who also co-supervises this project, is a co-author of this paper. No relationship existed between the other study participants and the interviewer prior to this study.

### Study Design

In line with the exploratory nature of the qualitative research strategy, semi-structured, open-ended, qualitative interviews were chosen. This research-tool allows to address a broad topic in a comparable, structured manner, with the structure of open questions defining the areas to be explored, while providing the space to develop further upon issues geneticists regard essential (Leech 2002; Weiss 1995). An interview guideline was developed based on available literature to explore medical geneticists’ views about the ethical issues in paediatric genome-wide sequencing (GWS) (supplement 2). It was created to be flexible allowing further exploration of issues which came up during the interview. Based on the first two interviews, it was decided that no further piloting or adaptation of the interview guides was necessary.

Participants were mainly selected through purposive sampling, combined with snowball sampling (Palinkas et al. 2015; Marshall 1996). Medical geneticists from two continental European countries (Germany and Switzerland) were included. Both countries share similar legal and practical conditions. In both countries, the ethico-legal guidelines delineate the counselling requirements for genetic testing as follows: Pretest counselling is obligatory for diagnostic testing, where the provision of genetic counselling must be presented as an option. Legal mandates stipulate that non-directive genetic counselling is a mandatory component for presymptomatic and prenatal testing, as well as for genetic testing related to family planning (§10 Abs. 2 GenDG; GUMG Art.21). In both countries medical geneticists are board-certified physicians. Furthermore, under the current legal and practical circumstances in Germany and Switzerland, medical geneticists are from the expert side the group mostly involved in the decision-making process, in genetic counselling and know best about the techniques and procedures; for example, the widespread recognition of genetic counsellors as a profession is only beginning under the condition that they work under the professional responsibility of medical geneticists (Filges et al. 2022; Schwaninger et al. 2021).

Participants were initially identified by searching through the websites of relevant hospitals and private institutions, as well as via personal contacts and recommendations from already identified the geneticists. They were contacted by email, and suitable dates for an interview were arranged with those willing to participate. One reminder e-invitation letter was sent to non-responders two to four weeks later. Overall, J.E. contacted forty-nine geneticists, of whom twenty agreed to participate (response rate 40.82 per cent).

We adopted a pragmatic approach in determining data saturation and critically evaluated theme saturation throughout the data analysis phase (Low 1019). Interviews were conducted between February 2020 and April 2021. Most interviews were held in German, few interviews in the French-speaking part of Switzerland (Romandy) were done in English. Only the participant and the researcher were present during the interview. All interviews were audio-recorded and had a mean duration of fifty-five minutes (range 29–71 minutes). They were transcribed verbatim, and transcripts were pseudonymized.

### Data Analysis

Using the interview transcriptions in their original language, J.E. and I.K. performed an inductive analysis of the data with the qualitative software MAXQDA employing reflexive thematic analysis (Braun and Clarke 2006; Braun and Clarke 2019). As laid out by Braun and Clarke (2006) in their seminal paper, thematic analysis usually follows six phases of analysis: a.) Familiarizing yourself with your data; b.) Generating initial codes; c.) Searching for themes; d.) Reviewing themes; e.) Defining and naming themes; f.) Producing the report. We followed these phases. A coding system consisting of 133 codes was created by discussing and comparing individually developed codes, coded segments, and writing memos: J.E. and I.K. coded three interviews independently, discussing their results after each interview and developing the coding framework. The remaining seventeen interviews were coded by J.E. on the basis of that coding framework, while IK double checked several interviews. New codes introduced, interpretative discrepancies, and any uncertainties were discussed between J.E. and I.K.

In an interpretive and iterative process, main themes on the overall subject of non-directiveness were generated and critically discussed and reflected with the other co-authors based on analytic reports written by J.E.

## Results

Twenty medical geneticists who work with children in Germany (*n*=10) and in Switzerland (*n*=10) (German-speaking part and French-speaking part) were interviewed; fifteen worked in academic hospitals and five in private specialty practices or private laboratories.

This article is part of a larger project that explores ethical considerations associated with genome-wide sequencing in paediatric patients. Here we present the results of one major theme that we found to be significant within the interviews, which is non-directiveness (other topics are published elsewhere such as Eichinger et al. 2023a, 2023b. The topic of non-directiveness was not explicitly included in the interview guide; however, all except two participants elaborated on it in detail during the course of the interview; e.g. when engaging in discussions about questions three, four, or seven of the interview guide (see supplement 1).

Although many brought up the issue of non-directiveness spontaneously, when probed on what the term actually means for them, participants’ responses revealed major uncertainties and divergences in how they conceptualized and used non-directiveness in practice.

### The Nature of Non-Directiveness

**Fig. 1:**
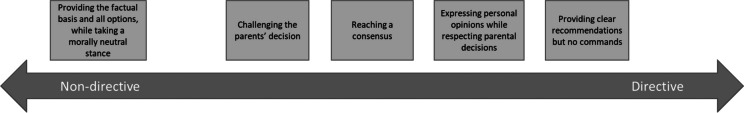
The spectrum of understandings of non-directiveness

Participants’ views on how non-directiveness should be understood fell on a spectrum (figure 1). At one end of the spectrum were the participants who defined non-directiveness as providing information in a neutral manner, then leaving the decision entirely to the patient or their parents.

*For me, [being non-directive] means that it is my job to offer the family or the patient information and to establish a basic knowledge for the patient. Or with the family, so that they can make the decision themselves. That means for me that it is the requirement or the desire for my consultation to choose as few value-judgmental words as possible, so as not to push the patient in one direction or another. [...] My goal is to present the situation and the data to the patient as neutrally as possible, so that they can then make the decision themselves. (DE19)*

It was stated that all available options had to be offered on an equal footing and that one should not hide any options even if they themselves did not approve of them. This included adopting a morally neutral stance and using as few value-laden words as possible so as not to push the patient or their parents in one direction or another. The statements of several medical geneticists indicated that this was not always easy, because one also holds one’s own—possibly strong—opinion. According to them, it was impossible to take a truly neutral stance on the matter itself but only to try not to show one’s own attitude to the patients or their parents. Furthermore, some emphasized that their definition of non-directiveness included not only not trying to change the patient’s/ parent’s minds but also expressing understanding, supporting, even encouraging them*.*

Other participants stressed that, while one should avoid providing recommendations, they thought it was important to make patients face the consequences and implications of possible decisions, to ensure that they were well thought out.

*So, this is why, when the people, they say no, I challenge them. […] But it really requires that you are sure of the counselling you gave the patient. (CH10)*

Other participants rather emphasized they aimed to find a joint decision and consensus with the patient, also regarding, for example, whether variants of unknown significance (VUS) and unsolicited findings (UFs) were communicated.

*No, I do not give a recommendation. I inform. I inform and then we come to a shared conclusion. (CH7)*

Other participants defined non-directiveness in such a way that allowed them to express their personal opinions but then still accept the decision of the patient/parents. For these participants it was acceptable to provide recommendations if the patient/parent asked for it, but that it was important to be aware of and articulate one’s own bias and that there was not always a clear right or wrong approach. One participant stated that he/she immediately made his/her opinion transparent because most parents would read an opinion from him/her regardless, even if he/she had none at all and by expressing his/her own opinion, there was at least the possibility to contextualize it.

*But if they asked me that directly, I would say this is what I would probably decide. I don’t want to withhold that from them, but that doesn’t mean that this is the best decision for them. And that I myself am not quite sure whether I would really decide that way if I were in their situation. Because I can’t picture it, but because I’ve never been in such a situation myself. So that’s how I would talk to them. But that’s simply five minutes that you have to take. That costs time, no one pays you for that. I wouldn’t withhold it. I don’t understand being non-directive as locking arms and not telling. (DE18)*

At the other end of the spectrum, some participants argued that while there must be openness to the outcome of the counselling, the principle of non-directiveness was still respected if only recommendations for action were provided but no commands for actions. These participants thought it was acceptance to “*steer*” parents to make the “*right*” decisions.

*As non-directive as possible, but you have to help them find the right way. You simply cannot teach people in this one hour everything that I am telling you now and that I have learned in twenty-five years of professional experience. [...] First of all, non-directive means that a genetic test is offered but not ordered. This already starts with the wording. Which we also adopt in the physician’s letters. And then there is maybe one specific thing, so it’s actually always the words, how you use them. You say ’is offered’. […] We would not actually say “the test is indicated.” Only “medically indicated.” Because “indicated” means you have to do it. And that, in turn, is decided by the patient. [...] Sometimes it is offered a bit more strongly, but in the end it is always the patient’s free decision. (DE20)*

### When Non-directiveness Is Appropriate

#### Non-directiveness is always appropriate

According to some participants, the requirement of non-directiveness should always be adhered to. They also explicitly referred to the question of whether the parents want to continue with the diagnostics of their symptomatic child. The reasons they provided why non-directiveness was also indicated regarding diagnostics were, for example, that it was often difficult for parents to deal with a diagnosis, especially if there were no courses of intervention and it was associated with a poor prognosis as well as justified concerns about data security.

#### Non-directiveness is not appropriate when too much is at stake

Several participants stressed that non-directiveness is not appropriate when too much is at stake. One participant went as far as saying that, in relation to cases such as tumours and spinal muscular atrophy, he/she could “no longer reconcile with my conscience” to be non-directive and would have “chosen the wrong profession” (DE11) because there might be therapy options. According to some participants, especially in time-critical situations the principle had to be dismissed, e.g. acutely ill newborns for whom rapid GWS could be extremely important, not only in order to not miss any therapy options but also not to subject the child to many more examinations and therapy attempts in the case of an unfavourable prognosis.

*There are differences. So, if it is the typical case that I have just described, then I have the feeling that we are not missing out on anything. Then I say that the probability that I can concretely help the child through my diagnostics is not very high. The child continues to develop within its scope, and the most important thing it needs is support and loving parents. I can understand that very well. Here we are also very non-directive in our counselling. Where we are directive is in acute cases such as the one described, a child with a metabolic abnormality on the ward, where we also say to the parents: “You know, the genetics in this case is the X-ray, the MRI, that serves to establish the diagnosis and, from our point of view, that must be done.” (DE15)*

#### Non-directiveness and unsolicited findings

The statements of the participants also differed with regard to the question of whether the principle of non-directiveness applied to questions concerning UFs. DE11 said that he/she was sometimes directive here and considered this appropriate. Others, however, emphasized that it was important to take a non-directive approach to UFs as well.

#### Non-directiveness and reproductive decisions

Several participants stated that reproductive decisions were the most crucial, or even only realm, where non-directiveness applied. In the domain of decision-making stated by several participants as the most difficult—whether to carry out termination of pregnancy or not—one should be unequivocally non-directive. When counsellees asked for advice one should always try to provide different perspectives and emphasize the personal nature of the decision. On the other hand, a participant said that terminations of pregnancy which he/she herself finds ethically unjustified are precisely the cases where he/she dropped her non-directive stance and clearly stated his/her opinion. Some participants described family planning situations in which they felt the need to redirect parental considerations towards the potential challenges of having an additional affected child in a long-term perspective.

*So, the only situation where I push a bit the decision, is when I sense that the parents want to have another child, or the mother is pregnant, and they have a child that is deficient, that is too small for the parents to have realized what it will imply, and that there is a possibility that there will be a second child affected, and the parents, they do not realize that the child, when it will grow, it will not be as easy as it is now with the small age. So, this is the only situation where I push a bit. (CH10)*

### Barriers to Non-directiveness

The concerns of many participants about the concept of non-directiveness were also reflected in the frequent statements that it was impossible to be non-directive anyway and it was only an “abstract goal” (DE19). Through the selection of information, choice of words, facial expressions, gestures, and other body language, one often revealed one’s personal opinion anyway and could hence also be directive. The other reason often stated for the impossibility of non-directiveness was that the principle simply overburdened too many patients/parents, it was even stated to be “cruel for the patient” (CH10). Most people were helpless with a non-directive approach. Precisely because the impact of the decisions was often so large, parents found it difficult to make decisions on their own.

*And these are also often such, yes, such ethical all-or-nothing issues. When you say you’re going to terminate the pregnancy, you’re deciding against life. And those are different realms than when you say, “We have to operate on the meniscus.” That’s just the way it is. It often makes it difficult for the patients. Because, of course, they are completely overwhelmed by having to make this decision. And one would probably feel the same way. (DE16)*

### Comparison With Other Areas of Medicine

Participants repeatedly brought up the question why non-directiveness was upheld to such a high degree in genetics compared to other disciplines. They mentioned three reasons: 1) the atrocious history of eugenics; 2) the reach and impact of the decisions is much greater, because the findings often affect the whole family and can also be predictive; 3) the (still often) missing therapeutic options that could follow the examination results which many participants in the interviews repeatedly emphasized to strongly distinguish genetics as a discipline from others . However, for several medical geneticists their discipline had undergone a “paradigm shift” (DE20) already and non-directiveness had lost significance. The reason cited was that the findings acquired could increasingly also have therapeutic consequences for patients and were becoming more and more relevant for clinical care.

## Discussion

To our knowledge, this is the first qualitative study to examine the practice and attitudes towards non-directiveness of medical geneticists in Western Europe. Participants brought up the concept with great naturalness. However, its actual meaning was understood in varying ways and causing significant uncertainty. This uncertainty is not only reflected in the various definitions mapped out here but also in the differing takes on when the principle should be applied, both ethically and legally. Several participants stressed that the principle was not feasible in practice anyway and was no longer ethically justified due to an increasing likelihood of therapeutic implications resulting from genomic testing outcomes. The insights provided by our empirical study accord with the ongoing theoretical debate regarding the definition, legitimacy, and feasibility of the principle (Resta 2006; Biesecker, Peters, and Resta 2019; Jamal, Schupman, and Berkman 2020; Chańska 2022; Koplin et al. 2022).

In the euro-western cultural context, where our study was situated, an individual interpretation of autonomy is predominantly accepted and a prevailing ethical principle. While we recognize that perceptions and the adherence to non-directiveness may vary in other parts of the world, the diverse understandings and practical applications of the principle we found within our interview sample make it hard to declare clear differences between the attitudes towards non-directiveness here and in, for example, English-speaking countries, where most of the aforementioned theoretical debate surrounding the principle originates. Our project involved geneticists from Germany and Switzerland, encompassing both, the French-speaking and German-speaking parts of Switzerland. Despite some interview participants suggesting a potential inclination towards reservation and fundamental scepticism towards genetic tests in Germany and the more Germany-oriented German-speaking part of Switzerland, attributed to Germany’s historical role in advancing eugenic programmes, our detailed analysis did not reveal any overarching data trends between the two countries or the language regions of Switzerland regarding attitudes towards non-directiveness. An adequately nuanced understanding and application of non-directiveness appears crucial. As genetic testing becomes more prevalent in healthcare, there are areas of practice where it is appropriate to deviate from non-directiveness, make recommendations and to steer a patient towards a specific course of action. This particularly applies to cases when genetic testing is expected to be useful in guiding medical interventions, especially in the treatment of patients affected by or at risk of familial cancer syndromes or inherited cardiac disorders (Clarke 2019). In such instances, the person-centred decision-making (PCDM) approach is appropriate—just as it is widely acknowledged in all other fields of medicine that healthcare has to be person-centred (Elwyn, Gray, and Clarke 2000; Bjerring and Busch 2021; Debrabander 2022).^2^ The PCDM framework places patients’ preferences and needs above those of healthcare professionals (HCPs), integrating the best available evidence to ensure optimal care tailored to each person. As such, PCDM combines the crucial elements of prioritizing patient needs as well as integrating evidence-based principles of care into the decision-making process (Weiner 2004; Vedam et al. 2019). The foundational idea of person-centred care is defined by active participation, an authentic and open patient-HCP-relationship, and taking into account contextual factors that influence care, e.g. the therapeutic environment, the language used and the patients’ wider value systems such as religious and cultural background (Institute of Medicine 2001; Kitson et al. 2013; Rost et al. 2022). The person-centredness of a decision, which denotes its quality, is determined by the degree to which it aligns with the carefully assessed needs, values, and expressed preferences of an informed patient and is subsequently put into practice (Sepucha, Fowler, and Mulley 2004).

The inappropriateness of non-directiveness in these cases aligns with the paradigm shift described above, namely that the results of genetic examinations increasingly have therapeutic consequences and thus genetic exceptionalism further loses its legitimacy (Evans and Burke 2008; Garrison et al. 2019; Jamal, Schupman, and Berkman 2020; Zimmermann et al. 2020; Clarke 2023). Medical genetics has expanded significantly to all medical fields of responsibilities in healthcare. This includes addressing and evaluating the potential risks associated with developing diseases and managing their complications. This particularly applies when the concept of “actionable results” is understood in a broader sense, also encompassing, for example, the positive effects for the patients—but also for the whole family system—when putting an end to the diagnostic odyssey, implementing improved preventive measures or influencing lifestyle decisions (Jamal et al. 2017; Stivers and Timmermans 2017). But even with a more narrow concept of clinical utility due to more and more therapies e.g. in oncology and cardiology through genomic results (Kikano and Kannakeril 2022; Mateo et al. 2022). Thus, it can be argued that in the instances described above genetic counselling should not be any more or less (non-)directive than counselling and decision-making in any other medical field.

However, there are other contexts where non-directiveness still has a place. This pertains to matters of reproduction and prenatal diagnosis, involving decisions about whether or not to undergo preimplantation and prenatal testing and for what purpose. It also includes predictive genetic testing, if medical interventions cannot improve the patient’s outlook before the disease’s clinical onset and/or if the condition is highly variable in its clinical course and outcome. In such contexts, it is generally inappropriate for professionals to offer recommendations due to the deeply personal and fundamental value-linked nature of these decisions (Elwyn, Gray, and Clarke 2000; Clarke 2023).

In these areas, it is nevertheless crucial that patients receive adequate support in their decision-making process when needed. The uncertainty surrounding the principle of non-directiveness among the medical geneticists interviewed can give rise to interpretations that are potentially unethical and even harmful for both patients and medical geneticists. This includes the interpretation that non-directiveness would mean pouring out information and then adopting a passive stance, which is also discussed as a

… confusion between non-directiveness and neutrality. […] With neutrality comes a danger of professional indifference and patient abandonment: the counsellor may be hoping not to pressure the patient, but s/he is experienced as unengaged, excessively detached from the patient and indifferent to them. […] [I]f patients feel abandoned rather than supported by the professionals, then there has clearly been a failure of professional practice. (Clarke 2017, 554).

By adhering to this unduly narrow and hollow notion of autonomy, the autonomy of patients is actually jeopardized rather than safeguarded.

Furthermore, it is crucial to recognize that information is not inherently neutral. The decision of whether to transmit specific information, along with the timing and manner of doing so, can convey powerful recommendations about future actions. The notion of value-neutral facts is problematic, as the assumption that values and facts have no influence on each other is fallacious. Personal perspectives are likely to be evident in the manner one approaches both the patient and the family’s condition. This can for example be seen in the emphasis on either the more severe or milder aspects of the disease, as well as the inclusion or exclusion of possibilities for future treatment. Also the way a risk estimate is presented conveys personal values and societal attitudes and policies towards illness and disability is also value-laden and influences supposedly factual information (Rentmeester 2001; Scully 2008; Löwy 2015; Clarke 2019; Biesecker, Peters, and Resta 2019).

The different views on when principle of non-directiveness actually applies and when it is not relevant are in Germany and Switzerland intertwined with the question of when formal genetic counselling is obligatory at all. The distinction outlined in the Swiss and German ethico-legal guidelines between ordering diagnostic testing, which requires the obligation for medical counselling and informed consent and the obligation to propose genetic counselling but not necessarily doing it, and prenatal or predictive genetic testing, for which genetic counselling is obligatory, does not seem to be as clear-cut in practice (§10 Abs. 2 GenDG; GUMG Art.21). For the required nuanced understanding and application of non-directiveness—so that this principle becomes neither a burden for medical geneticists nor for patients—training, supervision, and education of those involved in genetic counselling situations is essential (Clarke 2019). The fact that in Germany and Switzerland geneticists are physicians means that they are committed to the Hippocratic Oath and, specifically, to the medical ethos that often attributes a special significance to *do not harm*. Through clinical experience and direct patient contact, ideally, doctors have a holistic perspective on the well-being of patients and their families.

## Limitations

First, the reported qualitative results cannot be generalized, because the sample cohort was not a randomly selected representation of the target population and was relatively small in size. Nevertheless, these findings provide an exploration and understanding of an important subject and twenty interviews is commonly considered an acceptable size in qualitative research. Furthermore, after twenty interviews we reached a point of theoretical saturation regarding the attitudes expressed by the medical geneticists. Our chosen qualitative methodology was designed to inductively explore the overall topic of paediatric genome-wide sequencing and gather comprehensive data, rather than seeking statistical representativeness and generalizability. Second, we collected the data in the context of diagnostic paediatric genome-wide sequencing. Since the interview participants consistently mentioned the concept of non-directiveness we considered this recurrent theme of interest and value. Third, there is a possibility that our interview study might have attracted medical geneticists with a particular interest in ethical issues, potentially sharing a specific set of opinions or possessing a heightened sensitivity to ethical considerations.

## Conclusion

This study demonstrates that medical geneticists in Germany and Switzerland understand and apply non-directiveness in varying ways. The concept seems to cause distress since many medical geneticists stated that the principle was difficult to put into clinical practice and was no longer ethically justified given the increasing likelihood of therapeutic implications resulting from genomic testing outcomes. The insights provided by our qualitative empirical study accord with the ongoing theoretical debate regarding the definition, legitimacy, and feasibility of the principle. An adequately nuanced understanding and application of non-directiveness seems crucial to circumvent the risks inherent in the principle, while promoting patient autonomy and beneficence.

## Supplementary Information

Below is the link to the electronic supplementary material.

Supplementary file1 (DOCX 22 KB)

Supplementary file2 (DOCX 38 KB)

## Footnotes

1 In this article by *patient* we mean either the patient or his/her surrogate decision-maker(s).

2 While we are aware that the term “shared decision-making” is more commonly used to describe the decision-making process between patients and doctors, we believe that the concept of PCDM is more suitable for several reasons, e.g.: the decision-making itself cannot and should not be shared; to emphasize that the person with all her values, including nonmedical ones, is in the center, and to maximize her autonomy (Nieuwenhuijze et al. 2014; Childress 2017; Vedam et al. 2019).

## Declarations

### **Data Availability**

Since participants have not provided consent to share their data, study materials cannot be made openly available. However, we will share portions of the study material relevant to the manuscript upon reasonable request. Please contact the corresponding author.
