# Supplementary material for: Attitudes Towards Non-directiveness Among Medical Geneticists in Germany and Switzerland
Source: J Bioeth Inq. 2024 Jul 22;21(4):711–22. doi: 10.1007/s11673-024-10355-x (PMC11882704; doi:10.1007/s11673-024-10355-x)
Supplement: Supplementary file 1 — Supplementary file1 (DOCX 22 KB) [file 11673_2024_10355_MOESM1_ESM.docx]

### Supplement 1 Extended Methods Section

This article is part of a PhD project that explores ethical considerations associated with genome-wide sequencing in paediatric patients. Here we present the results of one major theme that we found to be significant within the interviews, which is non-directiveness (other topics are published elsewhere such as author citation 1, author citation 2).

Study documents were reviewed by the responsible ethics committee (Ethikkommission Nordwest- und Zentralschweiz; EKNZ). The EKNZ declared that the study does not fall under the Swiss Human Research Act (Art. 2) because no health-related data were collected and data were coded and pseudonymized. Also, interviewing health professionals does not require ethical approval in Switzerland. Hence, ethical approval was not needed. Nevertheless, the EKNZ issued a declaration of no objection (Req-2019-00467) confirming that the project fulfills the scientific and ethical standards for research with humans (Art. 51, Swiss Human Research Act). Written informed consent was obtained prior to data collection and was considered subject to renegotiating over time. An information sheet was provided explaining the nature of the project, including the right to withdraw or stop the interview at any moment. The methods of the study are presented in accordance with the Consolidated Criteria for Reporting Qualitative Research (COREQ) reporting guideline (Tong, Sainsbury, and Craig 2007).

### **Research Team and Reflexivity**

Interviews were conducted by the PhD student in biomedical ethics J.E. Most of the researchers involved have long-established experience with qualitative research. They have backgrounds in Bioethics (J.E., B.E., S.ML., I.K.), Medicine/ Medical Genetics (B.E., I.F., I.K.), Political Science (J.E.), and Theology (B.E.). One of the interview partners who also co-supervises this project, is a co-author of this paper. No relationship existed between the other study participants and the interviewer prior to this study.

Our study seeks to gain a better understanding of the challenges regarding genetic counselling in pediatric genome-wide sequencing in order to promote patient-centredness and reduce moral distress of clinicians involved, keeping in mind the limits of the medical system.

Within this exploratory research, an epistemological stance of critical realism is suitable. We are convinced that themes “are actively created by the researcher at the intersection of data, analytic process and subjectivity” (Braun and Clarke 2017, 594). Accordingly, our analysis does not pretend to uncover the objective truth that slumbers in the data. There is an observed social reality in the interviews relating to the experiences of geneticists, but this social reality necessarily includes the interviewer. The themes we developed are our interpretations of the data and are based on our positionality. Therefore, special attention was paid to reflexivity and rigour in the research process in line with the standards set in the consolidated criteria for reporting qualitative research (COREQ). The theoretical frameworks underpinning our study are empirical bioethics (Ives et al. 2018), person-centred and shared decision-making as well as related bioethical principles and concepts such as autonomy and informed consent.

### Study Design

Semi-structured interviews with clinical geneticists working with children in Switzerland or Germany were conducted. In line with the exploratory nature of the qualitative research strategy, we employed purposive sampling combined with snowball sampling to facilitate access to the participant group (Palinkas et al. 2015; Marshall 1996). Medical geneticists from two continental European countries (Germany and Switzerland) were included. In these countries medical geneticists are board-certified physicians. Both countries share similar legal and practical conditions. In both countries, the ethico-legal guidelines delineate the counselling requirements for genetic testing as follows: Pretest counselling is obligatory for diagnostic testing, where the provision of genetic counselling must be presented as an option. Legal mandates stipulate that non-directive genetic counselling is a mandatory component for presymptomatic and prenatal testing, as well as for genetic testing related to family planning (§10 Abs. 2 GenDG; GUMG Art.21). In these both countries medical geneticists are board-certified physicians. Furthermore, under the current legal and practical circumstances in Germany and Switzerland, medical geneticists are from the expert side the group mostly involved in the decision-making process, in genetic counselling and know best about the techniques and procedures; for example, the widespread recognition of genetic counsellors as a profession is only beginning under the condition that they work under the professional responsibility of medical geneticists (Filges et al. 2022; Schwaninger et al. 2021).

Geneticists were initially identified by searching through the websites of relevant hospitals and private institutions, as well as via personal contacts and recommendations from already identified geneticists. Electronic study invitation letters were sent to them. One reminder e-invitation letters were sent to non-responders 2-4 weeks later. Overall, J.E. contacted forty-nine geneticists out of which twenty agreed to participate (response rate 40,82%). For all who agreed to participate, a telephone or in-person meeting was scheduled based on geneticists’ preferences. Twenty geneticists who work with children in Germany (n=10) and in Switzerland (n=10) (German-speaking part and French-speaking part) were interviewed; fifteen worked in academic hospitals and five in private specialty practices or private laboratories. These numbers are justified by feasibility within the research project and are a commonly accepted size in qualitative research (Mason 2010). We adopted a pragmatic approach in determining data saturation and critically evaluated theme saturation throughout the data analysis phase (Low 2019).

Interviews were conducted between February 2020 and April 2021. Most interviews were held in German, few interviews in the French-speaking part of Switzerland (Romandy) were done in English. Only the participant and the researcher were present during the interview. An interview guideline was developed based on available literature to explore geneticists’ views about the ethical issues in paediatric GWS (Supplement 2). Based on the first two interviews, it was decided that no further piloting or adaptation of the interview guides was necessary. All interviews were audio-recorded and had a mean duration of 55 minutes (range 29-71 minutes). They were transcribed verbatim, and transcripts were pseudonymized. Transcripts were not returned to geneticists.

The research tool of interviewing was chosen, because this method enables access to the interior life of people, gaining an awareness of how they feel, think, interpret and behave – also due to the possibility to ask follow-up questions and to encourage the respondents to develop some of their aspects further (Weiss 1995). Thus, qualitative interviewing enables access to data that could not be disclosed by quantitative methods, and is especially suitable for investigations of sensitive topics (Gill et al. 2008) – such as ethical issues around paediatric genome-wide sequencing. Quantitative interviews provide high precision through standardisation, but this is often at the expense of the completeness or profoundness of the answers gained. Reduced standardisation was accepted in our qualitative interviews for the benefit of increased density of the answers obtained, providing a fuller understanding of the geneticist’s views (Weiss 1995). Semi-structured, open-ended, qualitative interviews were chosen to cover a broad topic in a comparable, structured way, the structure of open questions defining the areas to be explored, while providing the space to develop further upon issues geneticists regard essential (Leech 2002).

The interview guide explored a wide range of issues, e.g. 1) in which cases geneticists would offer genome-wide sequencing of children to parents 2), how they experience the pretest- consultation 3) general attitudes towards the ethical challenges and opportunities raised by genome-wide sequencing in paediatrics. It was created to be flexible allowing further exploration of issues which came up during the interview.

### Data analysis

Using the interview transcriptions in their original language, J.E. and I.K. performed an inductive analysis of the data with the qualitative software MAXQDA employing reflexive thematic analysis (Braun and Clarke 2019; Braun and Clarke 2006). A coding system consisting of 133 codes was created by discussing and comparing individually developed codes, coded segments and writing memos. In an iterative and interpretive process, main themes on the overall topic of non-directiveness were generated and critically discussed and reflected with the other co-authors based on analytic reports written by J.E.

**References**

Author citation 1: blinded for peer review

Author citation 2: blinded for peer review

Braun, V., and V. Clarke. Using Thematic Analysis in Psychology. *Qualitative Research in Psychology* 3 (2006): 77-101.

Braun, V., and V. Clarke. Reflecting on Reflexive Thematic Analysis. *Qualitative Research in Sport, Exercise and Health* 11, no. 4 (2019/08/08 2019): 589-97. https://doi.org/10.1080/2159676X.2019.1628806.

Ives, J., M. Dunn, B. Molewijk, J. Schildmann, K. Bærøe, L. Frith, R. Huxtable*, et al.* Standards of Practice in Empirical Bioethics Research: Towards a Consensus. *BMC Medical Ethics* 19, no. 1 (Jul 10 2018): 68. <https://doi.org/10.1186/s12910-018-0304-3>.

Gill P., K. Stewart, E. Treasure, B. Chadwick. Methods of data collection in qualitative research: interviews and focus groups. *British Dental Journal*. 2008;204(6):291-5.

Leech, B. L. Asking Questions: Techniques for Semistructured Interviews. *PS: Political Science &amp; Politics* 35, no. 4 (2002): 665-68. https://doi.org/10.1017/S1049096502001129.

Low, J. A Pragmatic Definition of the Concept of Theoretical Saturation. *Sociological Focus* 52, no. 2 (2019/04/03 2019): 131-39. https://doi.org/10.1080/00380237.2018.1544514.

Marshall, M. N. Sampling for Qualitative Research. *Family Practice* 13, no. 6 (1996): 522-26. https://doi.org/10.1093/fampra/13.6.522.

Mason, M. Sample Size and Saturation in Phd Studies Using Qualitative Interviews. *Forum Qualitative Sozialforschung / Forum: Qualitative Social Research* 11, no. 3 (08/24 2010). https://doi.org/10.17169/fqs-11.3.1428.

Palinkas, L.A., S. M. Horwitz, C. A. Green, J. P. Wisdom, N. Duan, and K. Hoagwood. Purposeful Sampling for Qualitative Data Collection and Analysis in Mixed Method Implementation Research.*Administration and Policy in Mental Health and Mental Health Services Research* 42, no. 5 (2015/09/01 2015): 533-44. https://doi.org/10.1007/s10488-013-0528-y.

Tong, A., P. Sainsbury, and J. Craig. Consolidated Criteria for Reporting Qualitative Research (Coreq): A 32-Item Checklist for Interviews and Focus Groups. *International Journal for Quality in Health Care* 19, no. 6 (2007): 349-57. https://doi.org/10.1093/intqhc/mzm042.

Weiss, R. *Learning from Strangers: The Art and Method of Qualitative Interview Studies.* New York: Simon and Schuster, 1995.
