# Supplementary material for: Attitudes Towards Non-directiveness Among Medical Geneticists in Germany and Switzerland
Source: J Bioeth Inq. 2024 Jul 22;21(4):711–22. doi: 10.1007/s11673-024-10355-x (PMC11882704; doi:10.1007/s11673-024-10355-x)
Supplement: Supplementary file 2 — Supplementary file2 (DOCX 38 KB) [file 11673_2024_10355_MOESM2_ESM.docx]

**Interview Guide**

Thank you very much for participating in our study! As you know, this interview is part of a research project of the Institute for Biomedical Ethics in Basel. I am currently doing my PhD there, this project is part of my dissertation.

Before we start, there is some more information that I need to share with you. As written to you, the aim of the project is to find out more about the experiences, needs and possible concerns with regard to genome-wide sequencing of medical geneticists and of young patients or their parents. For this reason, we will conduct interviews with parents of children who have received genome-wide sequencing and with medical geneticists who treat children. So the questions I ask to you will be with regard to exome/genome sequencing of children in the diagnostic setting.

Of course, the content of the interview will be kept strictly confidential. In the written transcript all mentioned names and places will be encoded so that no direct conclusions can be drawn about you.

If you wish not to answer to a question, please tell me so. Of course, you also have the right to interrupt or end the interview at any time without giving any reasons. It is also important for me to emphasize that this interview is really about your personal opinion, not about giving “right” or “wrong” answers.

Do you still agree to do the interview? *(... answer)*

Do you have any questions before we start? *( answer )*

Is it all right with you if I turn on the tape now*? (If so, turn on the tape recorder)*

1. Can you tell me about a specific case where you have offered exome or genome sequencing to a young patient or his/her parents?
2. And more generally, in which cases do you offer genome or exome sequencing to a patient or his/her parents?
3. How do you experience the pretest counselling processes?
4. How do you experience the decision-making process of the parents?
5. How do you deal with the results?
6. What is your opinion on the guidelines and legal regulations regarding genome-wide sequencing in Switzerland and Germany?
7. What do you think are ethical aspects, the pressing issues that need to be considered in relation to genome-wide sequencing? (If not already mentioned)
8. What is your opinion on prenatal genome-wide sequencing?
9. Anything else you want to add on the subject?
